# Machine learning approaches for diagnosing depression using EEG: A review

**DOI:** 10.1515/tnsci-2022-0234

**Published:** 2022-08-12

**Authors:** Yuan Liu, Changqin Pu, Shan Xia, Dingyu Deng, Xing Wang, Mengqian Li

**Affiliations:** Department of Psychosomatic Medicine, The First Affiliated Hospital of Nanchang University, No.17 Yongwaizheng Street, Donghu District, Nanchang 330006, Jiangxi Province, China; Queen Mary College, Nanchang University, Nanchang 330031, Jiangxi Province, China; Department of Internal Neurology, The First Affiliated Hospital of Nanchang University, Nanchang 330006, Jiangxi Province, China; School of Life Sciences, Nanchang University, No.999 Xuefu Avenue, Honggutan District, Nanchang 330036, Jiangxi Province, China; Clinical Diagnostics Laboratory, Clinical Medical Experiment Center, Nanchang University, Nanchang 330036, China

**Keywords:** EEG, artificial intelligence, psychiatric disorder, identification

## Abstract

Depression has become one of the most crucial public health issues, threatening the quality of life of over 300 million people throughout the world. Nevertheless, the clinical diagnosis of depression is now still hampered by behavioral diagnostic methods. Due to the lack of objective laboratory diagnostic criteria, accurate identification and diagnosis of depression remained elusive. With the rise of computational psychiatry, a growing number of studies have combined resting-state electroencephalography with machine learning (ML) to alleviate diagnosis of depression in recent years. Despite the exciting results, these were worrisome of these studies. As a result, ML prediction models should be continuously improved to better screen and diagnose depression. Finally, this technique would be used for the diagnosis of other psychiatric disorders in the future.

## 1 Introduction

Depression is a common mood disorder that has a substantial negative impact on the physical and mental health of patients [1,2]. The typical symptoms of depression encompassed low energy, fatigue, depressed mood, and even self-injurious or suicidal behavior in severe cases [3]. A recent survey from WHO has shown that the number of depression patients worldwide has exceeded 300 million people [4]. However, the clinical diagnosis of depression still relied on the Statistical Manual of Mental Disorders (DSM-V) and the subjective judgment of clinicians. Accurate identification and diagnosis of depression remained shrewd due to the lack of objective laboratory diagnostic criteria. Fortunately, the development of modern neurophysiological techniques offered a potential strategy for early disease detection. The application of the techniques in the field of clinical diagnosis has amassed large achievements in recent years.

Electroencephalogram (EEG) was widely used in neuroscience as a non-invasive neurophysiological technique. Compared to functional magnetic resonance imaging, EEG recordings had the advantage of shorter test times and lower prices, making them more suitable for identifying various psychiatric disorders [5]. Resting-state EEG (rsEEG) could accurately reflect the activity of human brain networks. Several studies have indicated that the frequency domain characteristics and functional connectivity (FC) of rsEEG were important in depression identification [6,7]. The analysis of rsEEG features might unravel the underlying complex neural mechanisms of depression. With the development of computational psychiatry [8], the use of rsEEG-based machine learning (ML) techniques to identify disease phenotypes has heightened increasing attention, which provided a theoretical basis for diagnosing clinical depression. Since Ahmadlou et al. first applied ML techniques to the early identification and diagnosis of depression [9], an increasing number of original studies have been published with exciting results [10,11,12]. Therefore, the rational application of rsEEG-based ML for diagnosing depression could help clinicians in rapid decision-making and treatment.

To systematically analyze the ML approaches for diagnosing depression using rsEEG, this study focused on reviewing the literature pertained to rsEEG-based ML for s depression diagnosis. (1) A total of 36 related articles were included by systematically searching domestic and international databases and filtering by specific criteria. (2) The ML approaches and their accuracy were highlighted in the studies above. Finally, this study would discuss the current status of rsEEG-based ML studies in the field of depression diagnosis and furnish further suggestions for future research.

## 2 Methods

### 2.1 Literature search strategy

Our study retrieved the results of domestic and international data from 1 January 2010 to 1 June 2022. The Chinese databases included Zhiwang, Wanfang, and Wipu, and the English databases encompassed PubMed, Web of Science, and Medline. Meanwhile, we utilized subject terms + keywords for the literature search, with the search terms: (“depression” OR “depressive disorder”) AND (“electroencephalography” OR “EEG”) AND ML. Finally, a total of 435 articles were involved in the analysis. In addition, this study further widened the number of articles analyzed by conducting reference back and hand searching, and a total of 449 articles were retrieved.

### 2.2 Inclusion and exclusion criteria

We further screened the literature for initial inclusion in the analysis based on the following criteria: (1) the main purpose is depression diagnosis; (2) the sample includes patients with unipolar depression and healthy controls; (3) rsEEG data as the data driver; (4) depression detection using ML; and (5) accuracy as the primary outcome. In addition, duplicates, conference papers, and literature for which full text was not accessible were excluded from this study. Finally, a total of 36 relevant articles that met the inclusion and exclusion criteria were entailed.

## 3 Results

**Figure 1:**
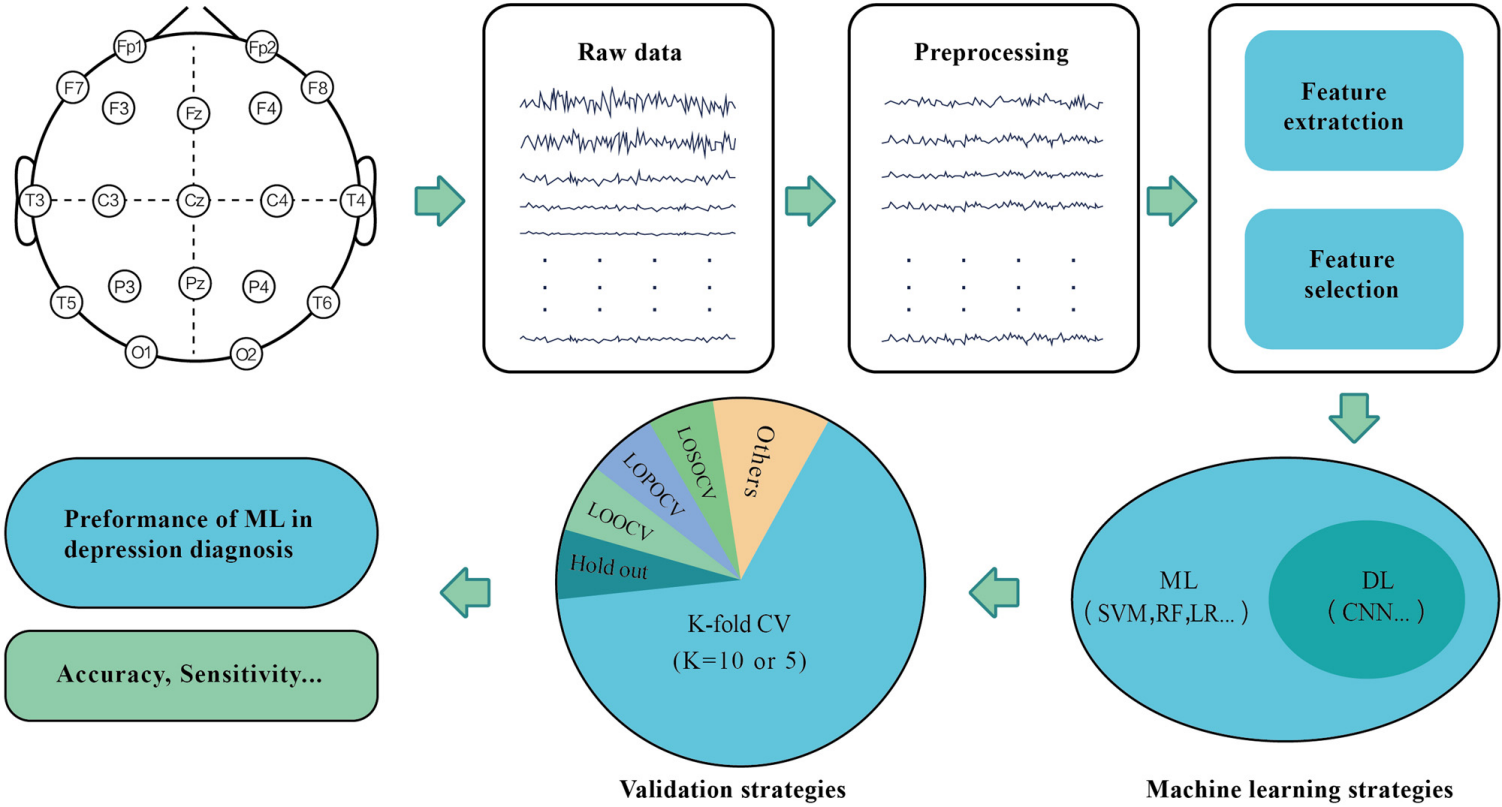
Overview of the EEG-based machine learning for depression diagnosis.

**Figure 2:**
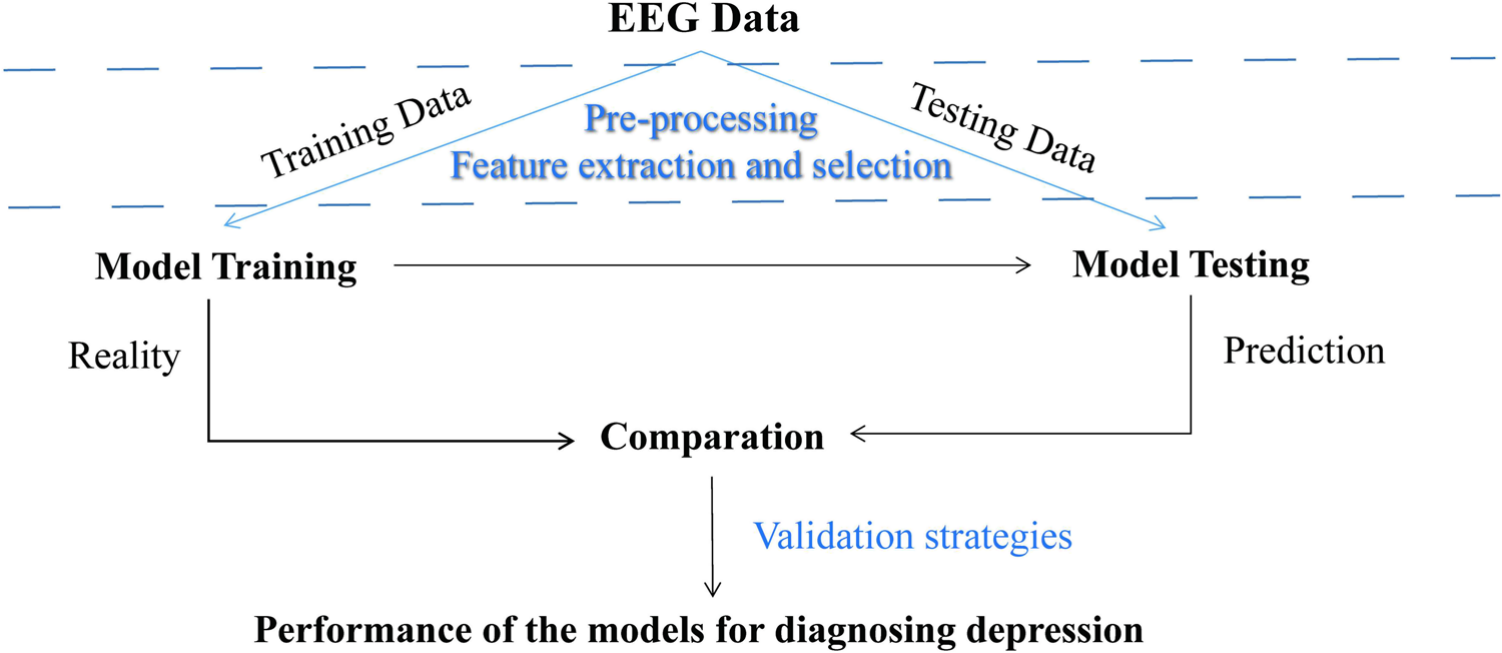
Flowchart of the EEG-based machine learning for depression diagnosis.

Our study systematically reviewed 36 articles on depression diagnosis published between 2010 and 2022 to illustrate the current value of rsEEG-based ML approaches in depression diagnosis. Because of the distinct methods used in different studies, our study would focus on the sample size, EEG data acquisition and preprocessing methods, feature extraction and selection, types of ML techniques, and their accuracy in depression diagnosis wielded in the aforementioned literature, as shown in Figures 1 and 2.

### 3.1 Sample size

Ahmadlou et al. published the first study based on rsEEG-based ML for the diagnosis of depression. In their study, a sample of 24 cases was included in the analysis [9]. Subsequently, Puthankattil and Joseph and Faust et al. increased the sample size (both 60 cases) and conducted similar studies to attain more credible results [10,12]. Further, Hosseinifard et al. published their study with a larger sample size (90 cases) [11]. Bairy et al. and Mohammadi et al., respectively, collected 60 and 96 cases for rsEEG-based ML on depression diagnostic analysis [13,14]. In the same year, Acharya et al. included 30 cases for analysis and used the data again in a subsequent study [15,16]. Later, Mumtaz et al. published four studies with sample sizes ranging from 60 to 64 cases [17,18,19,20]. Liao et al. in a study published included a sample of 24 cases [21]. One year later, Cai et al. and Wan et al. included 265 and 65 cases in their analyses [22,23]. In 2020, nine studies respectively compiled samples ranging from 32 to 92 cases for analysis [24,25,26,27,28,29,30,31,32]. An increasing number of researchers have conducted studies using data from previous or public samples [29,31,33,34,35,36]. Recently, some researchers revealed the sample size (20–400 cases) in their studies [37,38,39,40,41,42,43,44]. To our knowledge, 400 cases are the largest sample size to date. Overall, a total sample of 2,545 cases was included in this study. The distributions of the training and testing sets are shown in Table 1.

**Table 1:** Comparison of the machine learning approaches for diagnosing depression using EEG and its accuracy

Sample size (training/testing)	Electrodes/frequency/duration	Data preprocessing	Data feature	Machine learning strategies	Validation strategies	Accuracy	Reference
24 (75%/25%)	19/256 Hz/3 min	Wavelet analysis	^6^HFD, ^7^KFD	^45^EPNN	NA	91.30%	[9]
60 (33.3%/66.7%)	Fp1-T3; Fp2-T4/256 Hz/5 min	^1^WT, Filter	^8^WE	^46^RWE, ^47^ANN	NA	98.11%	[10]
90 (66.7%/33.3%)	19/256 Hz/5 min	Artifacts remove	Bands power, ^9^DFA, Higuchi, ^10^CD, ^11^LLE	^48^KNN, ^49^LDA, ^50^LR	^72^LOOCV	90%	[11]
60 (90%/10%)	Fp1-T3; Fp2-T4/256 Hz/5 min	Filter	^12^ApEn, ^13^SampEn, ^14^REN, ^15^EntPh	^51^PNN, ^52^SVM, ^53^DT, KNN, ^54^NBC, ^55^GMM, ^56^FSC	10-fold cross validation	99.50%	[12]
60 (90%/10%)	NA	^2^DTC	SampEn, FD, CD, ^16^H, LLE, DFA	DT, KNN, NBC, SVM	10-Fold cross-validation	93.80%	[13]
96 (70%/30%)	28/500 Hz/6 min	Filter, ^3^FFT, Min-max normalization	Spectral feature	DT	Hold-out cross-validation	80%	[14]
30 (NA)	Fp1-T3; Fp2-T4/256 Hz/5 min	Artifacts remove	FD, LLE, SampEn, DFA, H, ^17a^W_Bx, ^17b^W_By, EntPh, ^18^Ent1, ^19a^Ent2, ^19b^Ent3, ^20^DET, ^21^ENTR, ^22^LAM, ^23^T2	SVM	NA	98%	[15]
64 (90%/10%)	19/256 Hz/10 min	Noise reduction (^4^BESA software)	^24^SL	LR, NBC, SVM	10-Fold cross validation	98%	[17]
63 (90%/10%)	19/256 Hz/10 min	Noise reduction (BESA software)	Alpha interhemispheric asymmetry, Spectral power	LR, NBC, SVM	10-Fold cross validation	98.40%	[18]
60 (90%/10%)	19/256 Hz/10 min	Noise reduction (BESA software)	^25^MST, Distances between nodes, Maximum flow between nodes	SVM, LDA	10-Fold cross-validation	90%	[19]
63 (80%/20%)	19/256 Hz/10 min	Noise reduction (BESA software)	NA	^57^CNN, CNN + ^58^LSTM	10-Fold cross-validation	98.32%	[20]
24 (95.8%/4.2%)	30/500 Hz/NA	Artifact remove (NeuroScan software)	Spectral feature (common spatial pattern)	SVM	^73^LOPOCV	80%	[21]
30 (90%/10%)	Fp1-T3; Fp2-T4/256 Hz/5 min	Artifact remove, Normalization	NA	CNN	10-Fold cross-validation	95.49%	[16]
265 (NA)	Fp1; Fpz; Fp2/NA/30 min	Filter	Frequency and power, ^26^CO, CD, Shannon entropy, Kolmogorov entropy, Power spectral entropy	SVM, KNN, TD, LR, ^59^RF	10-Fold cross-validation	76.40%	[22]
35/30 (97.1%/2.9%)/(96,7%/3.3%)	Fp1; Fp2/256 Hz/8 min Fp1/512 Hz/8 min	WT, Filtering, Normalization	Time domain and frequency domain feature, Wavelet feature, Power spectral entropy, CO, ApEn, Wavelet entropy	KNN, RF, LDA, ^60^CART	LOPOCV	86.67%	[23]
30 (85%/15%)	Fp1-T3; Fp2-T4/256 Hz/5 min	Filter, Artifact remove	NA	CNN + LSTM	10-Fold cross-validation	99.12%	[33]
32 (NA)	64/500 Hz/4 min	Filter, Artifact remove, Re-reference, 5ICA	Strength of the weighted network, Average characteristic path length, Average clustering coefficient	SVM	LOOCV	82%	[24]
32 (NA)	64/500 Hz/4 min	Filter, Artifact remove, Re-reference, ICA	Spectral asymmetry, DFA	SVM	LOOCV	90.60%	[25]
43 (90%/10%)	19/1,000 Hz/3 min	Artifact remove	FD, SampEn	^61^MLP, LR, SVM, DT, RF, NBC	10-Fold cross-validation	97.56%	[26]
32 (90%/10%)	64/NA/3 min	Filter, ICA	Interhemispheric asymmetry, Cross-correlation	KNN, SVM, CNN	10-Fold cross validation	94.13%	[27]
92 (NA)	19/125 Hz/NA	Filter, ICA	NA	CNN	5-Fold cross-validation	92.66%	[28]
44 (70%/30%)	FT7; FT8; T7; T8; TP7; TP8/512 Hz;/15 min	Filter, Re-reference, ICA	Band Power, Interhemispheric asymmetry, Paired asymmetry, SampEn, DFA	SVM	Hold-out cross-validation	96.02%	[34]
60 (90%/10%)	19/256 Hz/5 min	Filter, Re-reference, ICA	Band Power, Inter-Hemispheric Theta Asymmetry	SVM, LR, NBC, DT	10-Fold cross-validation	88.33%	[29]
64 (90%/10%)	19/256 Hz/5 min	WT	Band Power, ^27^WPD, ApEn, SampEn,	^62^E-KNN, KNN, SVM, MLP	10-Fold cross-validation	98.44%	[30]
64 (80%/20%)	19/256 Hz/10 min	ICA, Normalization	Asymmetry matrix image	CNN	5-Fold cross-validation	98.85%	[31]
64 (90%/10%)	19/NA/NA	Filter, Re-reference, ICA	Statistical, Spectra, and Wavelet feature, DFA, Higuchi, CD, LLE, CO, ApEn, Shannon entropy, Kolmogorov entropy, ^28^FC	SVM, LR, DT, NBC, ^63^RB, ^64^GB, RF	10-Fold cross-validation	99%	[36]
64 (80%/20%)	20/256 Hz/NA	NA	NA	CNN	5-Fold cross-validation	99.08%	[35]
48 (NA)	34/500 Hz/3 min	Artifact remove (Brain Vision Analyzer software)	^29^DC, ^30^DTF, ^31^PDC, ^32^gPDC, ^33^eDC, ^34^dDC, ^35^ePDC, ^36^dPDC	^65^eMVAR, ResNet-50 + LSTM	5-Fold cross-validation	95.90%	[37]
400 (70%/30%)	32/500 Hz/1.5 min	ICA	Band Power, Coherence, HFD, KFD	KNN, LDA, SVM	5-Fold cross-validation	91.07%	[38]
20 (NA)	18/400 Hz/20 min	Artifact remove	Band Power, ^37^APV, ^38^SASI, HFD, ^39^LZC, DFA	SVM, LDA, NB, KNN, DT	10-Fold cross-validation	95%	[39]
138 (NA)	19/2,000 Hz/30 s	Normalization	Frequency domain feature	LR, SVM, RF, ^66^PL,			
^67^Adaboost, ^68^GBDT	10-fold cross validation	89%	[40]				
56 (NA)	64/1,000 Hz/8 min	Filter, Artifact remove	^40^PSD, LZC, DFA	SVM, LR, LDA	^74^LOSOCV	89.29%	[41]
64 (90%/10%)	19/256 Hz/5 min	Filter, ICA	SL	^69^LC-KSVD, ^70^CLC-KSVD	10-Fold cross-validation	99%	[42]
34 (90%/10%)	128/250 Hz/3 min	Filter, ICA, Normalization	PSD, ApEn, ^40^Kol, CD, CO, LZC, ^41^Per_en,^42^SVDen,^43^Renyi_spectral	^71^CMBE, SVM, RF	10-Fold cross-validation	92.65%	[32]
64 (NA)	32/500 Hz/4 min	Filter, ICA, Artifact remove	PSD, ^44^MPC	SVM	LOSOCV	83.91%	[43]
78 (80%20%)	32/1,024 Hz/4 min	Filter, ICA, Artifact remove	Microstate, Omega complexity	SVM	5-Fold cross-validation	76%	[44]

Notes: ^1^WT, wavelet transform; ^2^DTC, discrete cosine transform; ^3^FFT, fast Fourier transform; ^4^BESA, software, the standard brain electric source analysis software; ^5^ICA, independent component analysis; ^6^HFD, Higuchi’s fractal dimension; ^7^KFD, Katz’s fractal dimension; ^8^WE, wavelet entropy; ^9^DFA, detrended fluctuation analysis; ^10^CD, correlation dimension; ^11^LLE, largest Lyapunov exponent; ^12^ApEn, approximate entropy; ^13^SampEn, sample entropy; ^14^REN, Renyi entropy; ^15^EntPh, bispectral phase entropy; ^16^H, hurst exponent; ^17a^W_Bx and ^17b^W_By, weighted center of bispectrum; ^18^Ent1, normalized bispectral entropy; ^19a^Ent2 and ^19b^Ent3, normalized bispectral squared entropies; ^20^DET, determinism; ^21^ENTR, entropy; ^22^LAM, laminarity; ^23^T2, recurrence times; ^24^SL, synchronization likelihood, ^25^MST, minimum span tree; ^26^CO, C0-complexity; ^27^WPD, wavelet packet decomposition; ^28^FC, function connectivity; ^29^DC, directed coherence; ^30^DTF, directed transfer function; ^31^PDC, partial DC; ^32^gPDC, generalized PDC; ^33^eDC, extended DC; ^34^dDC, delayed DC; ^35^ePDC, extended PDC; ^36^dPDC, delayed PDC; ^37^APV, alpha power variability; ^38^SASI, spectral asymmetry index; ^39^LZC, Lempel–Ziv complexity; ^40^Kol, kolmogorov entropy; ^41^Per_en, permutation entropy; ^42^SVDen, Singu-lar-value deposition entropy; ^43^Renyi_spectral, chaotic time series analysis of the Rayleigh entropy; ^44^MPC, mean phase coherence; ^45^EPNN, enhanced probabilistic neural networks; ^46^RWE, relative wavelet energy; ^47^ANN, artificial neural networks; ^48^KNN, K-nearest neighbor; ^49^LDA, linear discriminant analysis; ^50^LR, logistic regression; ^51^PNN, probabilistic neural network; ^52^SVM, support vector machine; ^53^DT, decision tree; ^54^NBC, Naïve Bayes classification; ^55^GMM, Gaussian mixture model; ^56^FSC, fuzzy Sugeno classifier; ^57^CNN, convolutional neural network; ^58^LSTM, long-short term memory; ^59^RF, random forest; ^60^CART, classification and regression trees; ^61^MLP, multilayer perceptron; ^62^E-KNN, Enhanced K-nearest neighbor; ^63^RB, RusBoost; ^64^GB, GentleBoos; ^65^eMVAR, extended multivariate autoregressive; ^66^PL, Pseudo-labeling; ^67^Adaboost, adaptive boosting; ^68^GBDT, gradient boosting decision tree; ^69^LC-KSVD, label consistent K-SVD; ^70^CLC-KSVD, correlation-based label consistent K-SVD; ^71^CBEM, content based ensemble method; ^72^LOOCV, leave-one-out cross validation; ^73^LOPOCV, leave-one-participant-out cross validation; ^74^LOSOCV, leave-one-subject-out cross validation, and NA, not available.

### 3.2 EEG data acquisition methods

The number of electrodes, sampling frequency, and sampling duration altered slightly between studies, which might lead to different analytical results. Ahmadlou et al. first recorded rsEEG signals for 3 min with the eyes closed in depressed patients and healthy controls using 19 electrodes (10/20 standards) with a sampling frequency of 256 Hz [9]. A great number of following research employed the same number of electrodes and sample frequency, with only minor changes in sampling duration. With the development of EEG acquisition technology, many researchers have raised the sampling frequency to 500 Hz or higher. In recent years, some studies have used 64 electrodes EEG devices for recording depressed patients and healthy controls to enrich the reliability of rsEEG signals [24,25,27]. Furthermore, researchers were increasingly interested in region-specific EEG signals. Puthankattil and Joseph, Faust et al., and Acharya et al. have respectively investigated four electrodes (Fp1-T3), (Fp2-T4) in the left and right hemispheres, using a sampling frequency of 256 Hz [10,12,14,16]. Cai et al. obtained EEG signals in three electrodes (Fp1, Fpz, and Fp2) and six electrodes (FT7, FT8, T7, T8, TP7, and TP8) [22]. Later, Wan et al. compared the effect of singles from single electrodes (Fp1) and pairs of electrodes (Fp1 and Fp2) on the performance of rsEEG-based ML in the diagnosis of depression [23]. The sampling duration of these studies tended to be longer, up to 30 min (Table 1).

### 3.3 EEG data preprocessing

Because EEG data were very shaky, weak, and prone to interference, it was critical to preprocess them. There were various methods to remove data noise, among which most studies use manual methods or filters to remove noise. In addition, Independent Component Analysis (ICA) has pertained in several studies as a common technique for noise removal. Mumtaz et al. and Liao et al. used specific software to remove various types of artifacts from EEG signals [17,21]. It was worth noting that the removal of EEG noise could potentially lead to the loss of useful physiological signals. Therefore, this step needed to be performed with caution. Kang et al. eliminated the adverse effects caused by odd lead data by normalizing the EEG data so that each channel has a similar amplitude scale [31]. In addition, techniques such as Fast Fourier Transform (FFT), Discrete Cosine Transform, and Wavelet Transform (WT) were also applied for the preprocessing of EEG signals in the included studies (Table 1).

### 3.4 Data feature extraction and selection

Data feature extraction and selection were one of the most important steps in ML. Feature extraction refers to the extraction of linear or nonlinear features from EEG data. Feature selection was the further dimensionality reduction of the traits to remove redundant and irrelevant information. The methods of data feature extraction and selection used by different researchers vary. Ahmadlou et al. calculated Higuchi fractal dimension (HFD) and Katz fractal dimension (KFD) values as features using two different algorithms [9]. Puthankattil and Joseph used a multi-resolution decomposition of EEG signals and selected wavelet entropy as the feature [10]. Hosseinifard et al. used detrended fluctuation analysis (DFA) as one of the feature extraction methods and included band power, HFD, and other data as EEG features [11]. Faust et al. extracted nonlinear features by extracting suitable subbands from EEG signals and using the procured signals as algorithm inputs [12]. Bairy et al. incorporated various features into the analysis [13]. Mohammadi et al. published a study focusing on EEG spectral features [14]. Mumtaz et al. respectively allotted features such as synchronization likelihood (SL) and alpha interhemispheric asymmetry for analysis in their studies [17,18,19]. Liao et al. proposed a new EEG feature extraction algorithm called kernel eigen-filter-bank common spatial pattern (KEFB-CSP) [21]. Cai et al. selected various linear or nonlinear features and feature selection methods [22]. Wan et al. selected the best traits of EEG signal by using GA to boost the performance of EEG-based ML in differentiating depressed patients from normal controls [23]. Shen et al. applied spectral asymmetry index (SASI), DFA, and GA for EEG feature extraction and selection [24]. In the same year, Shen et al. used network parameters and their Area Under Curve (AUC) of EEG of depressed patients and healthy controls as characteristics for ML analysis [25]. Čukić et al. used HFD and sample entropy (SampEn) as the main features [26]. To improve the classification accuracy of ML, Duan et al. combined interhemispheric asymmetry with cross-correlation as EEG signal features to compensate for the lack of single trait information [27]. Mahato and Paul and Mahato et al. successively used Relief Algorithm and Multi-Cluster Feature Selection to select the best EEG signal features such as band power, interhemispheric asymmetry, and SampEn [29,34]. Saeedi et al. conducted further studies using features such as band power, approximate entropy, and SampEn [30]. In the same year, Kang et al. used EEG asymmetric matrix images as features for deep learning (DL) screening of depression [31]. However, in other studies of DL, researchers did not report the strategy used in EEG feature extraction and selection. Recently, Ghiasi et al. calculated the mean phase coherence (MPC) index as the brain FC feature [43]. Movahed et al. not only used FC as the main feature in the analysis but also proposed a novel algorithm sequential backward feature selection (SBFS) for the features selection to derive the optimal classifier model [36,42]. Wu et al. and Liu et al. successively used SBFS to select the optimal features in their studies [38,41]. As time goes by, assorted feature extraction and selection methods were widely applied in this field (Table 1).

### 3.5 ML strategies

The ML strategies utilized by different researchers vary, Ahmadlou et al. used Enhanced probabilistic neural networks (EPNN) to classify the EEG signals of normal and depressed individuals [9], while Puthankattil and Joseph used Relative wavelet energy and Artificial neural networks (ANN) to differentiate the data [10]. Hosseinifard et al. used K-nearest neighbor (KNN), linear discriminant analysis (LDA), and logistic regression (LR) classifiers in their study [11]. Faust et al. compared the performance of probabilistic neural network (PNN), support vector machine (SVM), decision tree (DT), KNN, Naïve Bayes classification (NBC), Gaussian mixture model (GMM) and Fuzzy Sugeno classifier (FSC) to obtain the best classification model [12]. Bairy et al. analyzed the performance differences of multiple classifiers such as DT [13], while Mohammadi et al. scrutinized only the classification performance of DT alone [14]. Acharya et al., respectively, used SVM and (Convolutional neural network) CNN for EEG signal classification [15,16]. Mumtaz et al. successively chose various traditional ML classifiers and DL classifiers to explore the best performance of EEG-based ML in depression diagnosis [17,18,19,20]. Liao et al. used SVM to build the prediction models [21]. Cai et al. published a study systematically analyzing the performance differences of classification strategies including SVM, KNN, DT, LR, and random forest (RF) [22]. Wan et al. used KNN, RF, LDA, and Classification and regression trees (CART) classifiers [23]. Shen et al. explored the performance of SVM in different EEG features [24,25]. Multilayer perceptron (MLP), LR, linear, and polynomial kernel SVM, DT, RF, and NBC were used to differentiate between normal controls and depressed patients in the study by Čukić’s study [26]. Similar to Liao et al., Mahato et al. also wielded SVM to build prediction models, and then, they compared the performance of three different kernel functions of SVM in classification [21,29,34]. Saeedi et al. also focused on the application of combined enhanced K-nearest neighbor (E-KNN) and EEG signals to diagnose depression [30]. Movahed et al. obtained the best ML framework by comparing the performance of various classifiers in distinguishing normal and depressed individuals [36]. Subsequently, they also presented dictionary learning approaches for automated MDD diagnosis [42]. Both past and present, SVM had been more widely employed in related studies [39,40,41,43,44]. Meanwhile, an increasing number of studies had used CNN or CNN + LSTM for EEG recognition of normal individuals and depressed patients (Table 1).

### 3.6 Validation strategies

Most studies enlightened how to assess the stability of the performance of the above ML models. K-fold cross-validation (*K* = 10 or 5) is one of the most commonly used methods, and a large number of studies use this method to assess the classification performance of ML. Some studies assessed the reliability of classification accuracy by leave-one-out cross-validation (LOOCV) and its variants. Only two of all included studies used hold-out cross-validation (Table 1).

### 3.7 Accuracy of various ML strategies in depression diagnosis

Ahmadlou et al. found that the HFD in the Beta rhythm was a more effective feature in distinguishing between normal and depressed individuals, and the classification accuracy of EPNN was 91.30% [9]. Puthankattil and Joseph used ANN to classify normal and depressed signals and obtained an accuracy of 98.11% [10]. Faust et al. showed that PNN was the better classifier in distinguishing normal and depressed patients’ EEG signals with a classification accuracy of 99.50% [12]. Hosseinifard et al. found that the combination of multiple nonlinear features was effective in improving the accuracy of the classifier with a maximum accuracy of 90% [11]. Bairy et al. obtained an accuracy of 93.8% using the SVM classifier [13]. Mohammadi et al. earned the best accuracy of 80% using the DT algorithm [14]. Mumtaz et al. respectively reported the higher diagnostic accuracy (SVM: 98%, SVM: 98.40%, SVM: 90%, and CNN: 98.32%) in their four studies [17,18,19,20]. Liao et al. applied KEFB-CSP features and SVM classifier with an average diagnostic accuracy of 80% [21]. Acharya et al., respectively, used SVM and CNN to identify EEG data of normal and depressed individuals with the accuracy of 98% and 95.49% [15,16]. Cai et al. obtained 76.4% accuracy using the DT classifier [22]. Wan et al. applied LOOCV, and its classification accuracy was 86.67% [23]. Zhu et al. found that classification accuracies of the CNN-LSTM model for the right EEG signals (99.12%) were higher than in the left hemisphere [32]. Shen et al. found that using strength, average feature path length, and AUC of average clustering coefficient as features can effectively improve the classification accuracy with a maximum classification accuracy of 82% [24]. In the same year, Shen et al. obtained the highest classification accuracy of 90.60% using multi-channel EEG based on GA screening [25]. Čukić et al. reported an average accuracy of 90.24–97.56% for each of seven ML algorithms in distinguishing EEG between normal and depressed individuals [26]. Duan et al. obtained the highest accuracy of 94.13% using the SVM classifier [27]. Uyulan et al. respectively reported 89.33 and 92.66% classification accuracies in the left and right hemispheres using MobileNet architecture [28]. Mahato and Paul and Mahato et al. successively used different feature extraction and selection, and the classification accuracy of the SVM classifiers were 96.02 and 88.33% [29,34]. Saeedi et al. used an enhanced KNN classifier to attain the highest diagnostic accuracy of 98.44% [30]. Kang et al. used DL to analyze EEG asymmetric matrix images with a classification accuracy of 98.85% [31]. Especially, Zhu et al. used EEG and other data to detect depression with an accuracy of 92.65% [32]. Movahed et al. used an radial basis function kernel-based SVM classifier and obtained an average accuracy of 99% [36]. Wang et al. used CNN to classify normal and depressed individuals with an accuracy of 99.08% [35]. Other studies also obtained higher accuracy for depression diagnosis [37,38,39,40,41,42]. Recently, some studies have begun to use SVM to classify subclinically depressed and normal individuals (the accuracy of 76 and 83.91%, respectively; Table 1) [43,44].

## 4 Discussion

In recent years, an increasing number of studies have combined EEG with ML for the diagnosis of depression with thrilling results. Among the included studies, the highest classification accuracy was up to 99.5% [12], which offers the potential strategy for screening and prevention of early clinical depression. Although the MRI-based ML studies have attracted a lot of attention over time [45,46,47], EEG-based ML has achieved better performance in depression diagnosis in terms of both cost and classification accuracy [5]. It was worth pointing out that, despite the high accuracy of such studies in distinguishing normal individuals from depression patients, additional studies are needed to confirm their reliability and variability. Among all past published studies, the overall classification accuracy ranged from 76 to 99.5% with a large variation. This reason might be related to the sample size, data collection and preprocessing methods, various feature combinations, and ML models wielded by different studies.The small sample size was a common problem faced by most of the current EEG-based ML studies. Of the 36 studies published from 2010 to date, only three studies had a sample size of more than 100 cases [22,38,40]. The problem of the limited sample size further constrained the diagnostic utility of EEG signals at the personalized level of depression, which might be one of the important reasons for the stability of classification accuracy. Although increasing the sample size for diagnostic accuracy was not necessary [22], it was essential to improve the sample size for analysis in order to make prediction models applicable to a huge population. With the publication of more studies in related fields and the improvement of public databases, a growing number of studies used more data volume for further analysis. This has addressed the above-mentioned issues to some extent [29,30,31,33,35,36]. However, the generalization of the results from public databases is limited due to the variability in data collection and data processing. As a result, it was essential to constantly improve the public databases. In the future, researchers needed to focus on the standardization and reproducibility of EEG data acquisition and processing processes. In addition, the distributions of the training and testing sets should be reported in the study, because they had a direct impact on the accuracy and clinical application of the obtained results.

Feature extraction and selection were the indispensable steps in ML. The use of appropriate features facilitated the overall performance of the prediction model. Although some researchers have demonstrated that utilizing raw EEG data for DL prediction models provides excellent performance (98.32%) [20], selecting EEG features of depression is a crucial strategy to improve the model’s diagnostic accuracy. Currently, a large number of studies have reported the variability in EEG between depressed and normal individuals [49,50]. In depression patients, the asymmetry of different rhythms in the left and right hemispheres is one of the most valuable neurophysiological indicators [17]. Similarly, graph theory analysis based on FC has been widely used in the study of depression abnormalities [31]. Furthermore, various nonlinear characteristics have been widely used in ML models, outperforming various linear features in the diagnosis of depression [12,13]. It was worth noting that GA was used to reduce the dimensionality of the features to improve the performance of the classifier in some studies [14,24,25]. Therefore, it was mandatory to select and combine various EEG features in a rational way to further improve the accuracy of the ML model, especially the left and right hemisphere asymmetry of the alpha rhythm, FC, and various nonlinear traits.

Most studies used SVM and its variants as the main classifier, which may be related to its reliable theoretical foundation and flexible response characteristics to high-dimensional data [12,13,36,44,45]. SVM was used to classify EEG nonlinear features of depression with good accuracy in almost half of the past studies. However, the studies are also restricted by issues such as the small sample size and excessive nonlinear features, which may further lead to the overfitting of the data. It was reported that leave-one-out cross-validation or *k*-fold cross-validation applied could be avoided overfitting of the prediction model and thus improve the generalization ability of the model [49]. Unfortunately, some studies did not report explicit internal or external validation information, thus failing to ensure the reliability of the prediction model accuracy. Therefore, it was key to accurately select the appropriate ML method, data properties, and reasonable validation methods in the future. At present, DL, especially CNN, is gradually applied to a depression diagnosis. The self-learning functions of CNN can effectively obtain and integrate valid information from complex data to obtain better prediction ability (95.96%) [16]. Therefore, the application of DL to assist in depression diagnosis is the focus of ensuing research.

Diagnostic heterogeneity of depression might be one of the motives to lead the different results [48]. The different diagnostic tools and criteria might be used in the different studies about EEG-based ML of depression diagnosis. It could influence the performance of the classifier [43,44]. Furthermore, depression is usually co-morbidity with other mental disorders such as anxiety, substance use disorders, and borderline personality disorder [51,52]. Meanwhile, it was also difficult to distinguish between bipolar and unipolar depression [53]. EEG information might be different in various psychiatric symptoms. Fortunately, some studies have begun to employ EEG-based classification models to identify various depressive symptoms [54,55]. Especially, the researcher found that the resting-state connectivity biomarker could be used to define neurophysiological subtypes of depression [56]. It would be an important reference to precisely identify clinical subtypes of depression.

In conclusion, it was necessary to continuously optimize ML prediction models. To move the diagnostic window of depression forward and effectively prevent the onset and progression, some strategies should be adopted such as increasing the sample size, combining multiple EEG features, and using the DL model. In the future, it would benefit many patients with psychiatric disorders and high-risk groups, especially with affective spectrum disorders.

At the same time, the limitations of our article still needed to be deemed carefully. Our study only discusses using a single EEG signal as a data-driven ML model in the diagnosis of depression, which lacks clinical utility and accuracy compared to current ML models combining multimodal data. Given these limitations, we would further integrate socio-epidemiological survey data, neurobiological and molecular biology techniques, and other multimodal data to build more accurate artificial prediction models, which would eventually provide new strategies for early diagnosis of depression as well as other psychiatry disorders.
